# Mental health social care: scoping and developing new and necessary health and care research system

**DOI:** 10.1192/bjb.2025.25

**Published:** 2026-02

**Authors:** Adam O’Neill, Catherine A. Robinson, Duncan Tree, Michael Clark

**Affiliations:** 1 School of Health Sciences, University of Manchester, UK; 2 Association of Mental Health Providers, London, UK; 3 Care Policy and Evaluation Centre, London School of Economics and Political Science, UK

**Keywords:** Mental health social care, mental health, social care, health and care research system, mental health services

## Abstract

Mental health social care is an emerging and evolving field of practice and research within mental health care in the UK. It recognises the significant role played by social determinants in the development of mental illness and distress, and in recovery and well-being. By considering mental health social care as a distinct health and care research system, this paper outlines key priorities for research, funding and capacity building. It argues that mental health social care should be an essential component of mental health service delivery, and calls for a move towards holistic, person-centred care that addresses the social determinants of mental health, alongside biological and psychological factors.

Mental health social care is an emerging and evolving field of practice and research within mental health care in the UK. It recognises the significant role played by social determinants in the development of mental illness and distress, and in the recovery and well-being of those affected. Despite its importance, social care in mental health remains marginalised, underfunded and poorly integrated within broader mental health services and research. This paper argues for the recognition of mental health social care as a distinct field essential for addressing the complex social aspects of mental health. Using Sadana and Pang’s^1^ four-function model of a health research system, we appraise the current state of mental health social care and discuss the potential for further development of the system towards one capable of translating research into practice and enhancing mental health service provision.

## Defining mental health social care

Attempts to define mental health social care are complicated by the fact that there is no agreed upon universal definition of social care,^2^ and no definition is prescribed in relevant legislation.^3^ Nonetheless, in England, the concept of social care has become inextricably linked to the contents of the 2014 Care Act,^4^ which stipulates how and when social care should be provided by local authorities.^5^ Derived from this legislation, social care is widely conceptualised as including support for activities of daily living; personal care; mobilising; nutrition; medication; social interaction; maintaining relationships; involvement with wider society; personal safety; maintaining independence and dignity; and upholding human rights.^6–9^

In the context of mental health, social care exists to support people whose mental illness or distress impacts them across the above domains.^10^ Mental health social care, therefore, is intrinsically defined by a social understanding of health and well-being, drawing on frameworks and practice models that stem from a biopsychosocial approach.^11^ While acknowledging the importance of the biological and psychological aspects of mental illness and/or distress, mental health social care places the social on an equal footing with the bio and psycho, both of which have, traditionally, been more prominent in research and practice.^12^ Accordingly, where appropriate, mental health social care promotes adherence to the principle of least restrictive practice^13,14^ and prioritises non-institutional, community-based care and support. Promoting independence is core to the ethos of mental health social care, which recognises that recovery from mental illness or distress is a complex, non-linear and multidimensional process, as is the aetiology of mental illness or distress.

For recovery, all elements of a biopsychosocial understanding must interlink and work holistically.^10^ Practically, this means social care systems across the statutory, voluntary, community and independent sectors being central to recovery support. Local mental health systems spanning acute, community, social care and support services must remain alongside people experiencing mental health difficulty over the medium and longer term.^10^ This continuous support aims to help individuals cultivate a sense of identity, agency and belonging within their communities.^10^ It is also aligned to research on mental health social work that identifies that people in need of support for mental distress value most highly the fact that social workers ‘think about [their] whole life, not just [their] illness’.^15^

Mental health social care views and responds to mental illness and distress in light of their social and economic determinants, locating a person’s mental health difficulty within the world they inhabit, rather than exclusively their head. As such, mental health social care can be seen to have a distinct practice base, with application for those in the population who may require care and support and have related mental health needs: for example, people who are homeless or those dealing with substance misuse. For such people, psychoactive pharmacological intervention and a fixed-term therapeutic offering risk stumbling past the socioeconomic and circumstantial factors that also contribute to their mental distress.

Reflecting this notion, the Mental Health Social Care Policy and Oversight Group^10^ defines mental health social care as something that:

‘Empowers people living with mental illness, people experiencing mental distress, their unpaid carers, and local communities. It seeks to enable people to lead fulfilling and independent lives by providing information, advice and offering practical, personalised support with everyday activities. It facilitates agency and the ability to access a life with purpose, meaning and a voice as an active citizen – not just the absence of symptoms. Through working in and with communities, mental health social care helps to develop their capacity to be supportive, resilient and emotionally healthy.’

Attendees at a recent summit on the development of mental health social care research capacity felt that this was captured neatly in the aspiration that practitioners, peers, services and organisations improve lives by ‘being alongside people’.^16^

## The neglect of social care in mental health

Across mental health service provision, social care has often been overlooked and underfunded.^17^ Clinical interventions receive prominence in research and practice, yet the social aspects of care, such as support with housing, employment and community integration, are also crucial for holistic recovery and well-being.^18–20^ However, despite the opportunities provided by socially situated interventions, both for improving population mental health and reducing the risk of mental illness associated with social inequalities,^21^ in England, primary and secondary healthcare have become the default route for people experiencing mental distress.^22^ This sidelining of social care not only undermines mental health service efficacy, but might also exacerbate disparities in access and outcomes, particularly for marginalised communities.^23^

Poole and Huxley^12^ have critiqued the current state of the social–mental health care interface, noting that it is ‘mostly not social at all’. In the context of mental health, they argue that social care is often restricted to ‘very limited provision at arm’s length in the voluntary sector or institutional placement for people with chronic ill health’.^12^ This approach fails to deliver the comprehensive support systems needed to address the complex social determinants of mental health, such as housing, employment and community integration.^19,21^ Despite recognition from within community mental health teams (CMHTs) of the essential role played by social workers in providing holistic support,^24^ research has shown that social workers and their colleagues feel that current operational models prohibit a fully integrated approach to person-centred care.^24^ Social workers’ expertise in managing issues such as poverty and social isolation is critical, but their relations to colleagues within CMHTs are more ‘mechanistic’ than integrated, due to reduced operational capacities. One CMHT colleague described their contact with social workers as being when ‘directed or needed as opposed to that constant presence and awareness and involvement’,^24^ highlighting the current peripheral role of social care in mental health service provision.

This particularly affects those with complex needs, including from minority ethnic backgrounds, who might not trust healthcare systems or face cultural and linguistic barriers to access.^25^ The Department of Health and Social Care’s Independent Review of the Mental Health Act^23^ further emphasises that current mental health services are failing minority ethnic communities. This is partly a result of the systemic neglect of social care, which is vital for addressing unique challenges such as cultural insensitivity and inadequate support structures.^23^ People from minority ethnic backgrounds are disproportionately affected by adverse social determinants of health,^20,26^ yet the lack of robust social care frameworks means that current health and care systems are ill equipped to rise to this challenge.

Overall, this points to a need for a significant change in mental health service provision, with the indispensable role of social care in mental health being acknowledged. To begin this process, mental health social care should be recognised as a necessary, new research system.

## Mental health social care as a health and care research system

Sadana and Pang^1^ define a health research system as:

‘The people, institutions, and activities whose primary purpose is to generate high quality knowledge that can be used to promote, restore and/or maintain the health status of populations.’

They theorise that a health research system has four functions:^1,27^

- Stewardship: setting a clear vision and strategy for health research, defining and prioritising research areas and their evaluation and establishing/monitoring ethical standards.
- Financing: focusing on securing financial resources and ensuring the efficient and accountable allocation of those resources to various research initiatives.
- Creating and sustaining resources (capacity): developing and maintaining the necessary human and physical resources for conducting research.
- Producing and using research: generating high-quality, validated research outputs and effectively translating and communicating these findings to inform public policy, clinical practice and public opinion.

Clark and Chilvers^28^ have previously used this four-function model to assess England’s mental health research system. We propose that mental health social care should be evaluated via the same method as a health *and care* research system in its own right. Although social care has deep roots in mental health, mental health social care is a relatively new concept and under-researched and hence it is important from the outset to undertake this work to be able to plan its development. Therefore, for each function, we assess the current state of mental health social care as an emerging research system and explore how it can evolve into an effective, established system.

### Stewardship

The Department of Health and Social Care’s Framework for Mental Health Research^29^ acknowledges that ‘there is a need to involve organisations beyond traditional mental health services’ in research. It sets a vision for mental health research in the UK that recognises the need to build capacity with local authorities and the voluntary, community, faith and social enterprise (VCFSE) sector as key stakeholders. While the framework establishes a relevant research priority of emerging interventions and alternative settings, and highlights the necessity for a more diverse research community to facilitate research in these areas, it is unclear on how these goals should be developed and how the delivery of the vision will be monitored and evaluated. Nonetheless, the importance of a clear ethical framework is emphasised and key areas where the health–social care interface might be developed are identified, specifically: linking data-sets; joint studies across the social–health interface; infrastructure to support joint working practices; and the removal of unnecessary bureaucracy for researchers.^29^

Additionally, several emerging research priorities have been proposed by key stakeholders in mental health social care.^16^ These include developing an economic case for mental health social care; improving the interface of primary care and mental health social care; supporting approaches that help people to lead better lives; and adopting mental health social care approaches to prevention and advocacy. To deliver on these research priorities, stakeholders also made recommendations as to the make-up, structure and desired approach of a mental health social care research system. Specifically, they suggested that a pluralist perspective should underpin the research system, which would actively seek to include different voices and sections of society^16^; the system should also be methodologically inclusive and should provide support for existing networks of organisations and individuals working with and within marginalised communities; it should create environments that allow researchers to thrive; strengths-based^30^ perspectives should underpin research; and efforts should be made towards better understanding of outcomes relevant to mental health social care.^16^

Stewardship therefore appears to be evolving in the right direction. However, improved coordination among stewards, building on their emerging visions to plan, implement and monitor developments, will be a constructive next step.

### Financing

Overall, mental health social care is gaining traction at a time when the research funding landscape is favourable. The National Institute for Health and Care Research (NIHR) has significantly increased investments in mental health research, social care research and research with and within underserved communities.^31–33^ This boost in funding is a promising step. It provides much-needed resources to explore non-clinical interventions and alternative care settings, which are crucial for developing more effective mental health social care solutions aligned to the vision of mental health social care set out in the previous section.

Nonetheless, other funders need to commit to supporting this vision. At present, some research projects and topics may fall between the gaps of different NIHR funding streams that are rigidly attached to a particular focus. Given the nascent standing of mental health social care and the relative recency of this shift in funding prioritisation by the NIHR, there remains an opportunity for other funders to support important projects that would otherwise remain unrealised. For mental health social care to flourish, a concerted effort is needed to harmonise funding and research initiatives across funders, to create a cohesive and effective approach towards advancing mental health social care.

Although the financial function of the system is now appearing healthier, there are opportunities for stewards to work collaboratively with mental health social care stakeholders to continue to develop the financial systems to best deliver the vision for mental health social care research.

### Creating and sustaining resources/capacity

The National Workforce Stocktake of Mental Health Social Workers in NHS Trusts^34^ reported that 6584 social workers were employed in the mental health sector in England and Wales. However, in contrast to their clinical colleagues, social workers are not close to research.^35^ To bridge the gap between practice and research, the Research Advisory Group for the Chief Social Worker for Adults^36^ provided a framework of commitments towards engagement with research and using research evidence. This framework was designed for leaders in adult social work, social workers and their managers, educational leaders and guardians of the research system; it was endorsed by people with lived experience of mental health social care, and its contents were agreed to by key system stewards including the NIHR, Association of Directors of Adult Social Services (ADASS) and the British Association for Social Work and Social Workers (BASW). Specifically, the Charter proposes a framework that emphasises the importance of collaborative research partnerships among practitioners, researchers and service users to ensure that research directly improves practice and outcomes.^36^ It advocates for building research capacity and skills through investment in training opportunities for social workers, while emphasising the need for ethical and inclusive research practices that reflect the diversity of the population served by adult social care.^36^

While detailed intelligence about staffing in the VCFSE sector is more limited, we can be confident in asserting that the sector overall is even further removed from research than social work. Increasingly, although funding is available to support practitioners engaging in/with research,^37^ there remains a lack of dedicated funding for VCFSE research.^38^ Other barriers faced by VCFSE organisations include a lack of access to/familiarity with academic processes such as ethical approval procedures; costs of involvement and payment processes; limited access to current research – for example, in subscription-only journals; and the time and capacity commitments required.^38^ To increase access to, and awareness of, research opportunities in the VCFSE sector, Harlock et al^38^ recommend engaging VCFSE organisations earlier in the research process; providing seed funding for VCFSE involvement in research design; making co-production meaningful; creating spaces for relationship development and partnership-building activities; providing research training and additional support for VCFSE organisations; promoting and developing genuinely accessible findings; and taking steps to develop the research readiness of the VCFSE sector.

For lived-experience researchers, engagement is similarly complex. While undoubtably there has been an increased recognition of the value of what is often termed patient and public involvement and engagement,^39^ and the expertise of people with lived experience is afforded significant weight in research and funding applications,^40^ roles for lived-experience researchers are nonetheless predominantly freelance, ad hoc, short-term contracted and/or low paid.^41^ As such, individuals with lived experience who wish to engage with research enter an ill-defined career structure. Positive moves have been made to ensure that lived-experience researchers are ‘embedded’ throughout studies,^42^ and co-production is now highly encouraged^43^; however, there remain structural barriers to people with lived experience spearheading mental health research, especially without retraining in traditional academic settings.

For capacity building, it would be constructive to bring related research under the single banner of mental health social care, helping make evidence more clearly relevant and identifiable. There has already been outstanding work, including towards understanding social interventions for mental illness^44^; nonetheless, without a single, coherent identity, the impact of this work risks remaining diffuse and obscured by more organised biomedical and psychological research. Addressing this is one of the aims of the NIHR-supported Mental Health Social Care Incubator, which will build mental health social care research capacity by undertaking activities to help people with diverse research, and lived and practice experience, develop their engagement with mental health social care research and practice.^45^ The Incubator’s initiatives include a ‘Stepping into Research’ programme that supports people with mental health social care research interests towards transforming their ideas into competitive grant applications.^46^ The Incubator will also act as a central hub for a growing network of mental health social care researchers, organising summits (e.g. see Mental Health Social Care Incubator^16^) and linking researchers with shared interests.

Overall, there are welcome developments for developing research capacity in mental health social care. Nonetheless, opportunities remain for stakeholders to consolidate the lessons from these developments and work to accelerate capacity development. Without a concerted focus on this, capacity development will be very gradual, hampering the growth of the mental health social research system.

### Producing and using research

The production and translation of research into practice is largely dependent on the success of the three functions previously described. For mental health social care, this is presently an evolving process from a relatively low base. With limited investments to date in building research capacity in mental health social care, there is likely to be less awareness of, interest in, or capability to engage with, research evidence to drive service improvements. Nonetheless, we have seen through our activities to date a widespread enthusiasm for engaging more with research, by both conducting it and using evidence to inform practice improvements.

Enhancing the profile of mental health social care in research, and the resultant greater awareness of research in mental health social care, have some solid foundations. However, focused attention is needed to make the most of this grounding and ensure translation of research evidence into better outcomes for people. This is arguably the most important step for mental health social care. Building on the ‘integration white paper’,^47^ the Mental Health Social Care Policy and Oversight Group^10^ suggest that integration of local systems should be the vehicle through which this is achieved. They make multiple recommendations around the commissioning and successful integration of mental health social care into current mental health and social care systems, several of which align with the evaluations and conclusions reached in this paper.

In conclusion, this paper highlights the need to recognise mental health social care as an essential component of mental health service delivery. It calls for a shift towards holistic, person-centred care that addresses the social determinants of mental health alongside biological and psychological factors. By considering mental health social care as a distinct health and care research system, the paper outlines key priorities for research, funding and capacity building.

## Data Availability

Data availability is not applicable to this article because no new data were created or analysed.
